# Multi-omics approach to study the growth efficiency and amino acid metabolism in *Lactococcus lactis *at various specific growth rates

**DOI:** 10.1186/1475-2859-10-12

**Published:** 2011-02-24

**Authors:** Petri-Jaan Lahtvee, Kaarel Adamberg, Liisa Arike, Ranno Nahku, Kadri Aller, Raivo Vilu

**Affiliations:** 1 Tallinn University of Technology, Department of Chemistry, Akadeemia tee 15, 12618 Tallinn, Estonia; 2 Competence Center of Food and Fermentation Technologies, Akadeemia tee 15b, 12618 Tallinn, Estonia; 3 Tallinn University of Technology, Department of Food Processing, Ehitajate tee 5, 19086 Tallinn, Estonia

## Abstract

**Background:**

*Lactococcus lactis *is recognised as a safe (GRAS) microorganism and has hence gained interest in numerous biotechnological approaches. As it is fastidious for several amino acids, optimization of processes which involve this organism requires a thorough understanding of its metabolic regulations during multisubstrate growth.

**Results:**

Using glucose limited continuous cultivations, specific growth rate dependent metabolism of *L. lactis *including utilization of amino acids was studied based on extracellular metabolome, global transcriptome and proteome analysis. A new growth medium was designed with reduced amino acid concentrations to increase precision of measurements of consumption of amino acids. Consumption patterns were calculated for all 20 amino acids and measured carbon balance showed good fit of the data at all growth rates studied. It was observed that metabolism of *L. lactis *became more efficient with rising specific growth rate in the range 0.10 - 0.60 h^-1^, indicated by 30% increase in biomass yield based on glucose consumption, 50% increase in efficiency of nitrogen use for biomass synthesis, and 40% reduction in energy spilling. The latter was realized by decrease in the overall product formation and higher efficiency of incorporation of amino acids into biomass. *L. lactis *global transcriptome and proteome profiles showed good correlation supporting the general idea of transcription level control of bacterial metabolism, but the data indicated that substrate transport systems together with lower part of glycolysis in *L. lactis *were presumably under allosteric control.

**Conclusions:**

The current study demonstrates advantages of the usage of strictly controlled continuous cultivation methods combined with multi-omics approach for quantitative understanding of amino acid and energy metabolism of *L. lactis *which is a valuable new knowledge for development of balanced growth media, gene manipulations for desired product formation etc. Moreover, collected dataset is an excellent input for developing metabolic models.

## Background

*Lactococcus *(*L*.) *lactis *is the most intensively studied lactic acid bacterium and it has a great industrial importance. In addition to its wide usage in the dairy industry, *L. lactis *subsp. *lactis *IL1403 was the first lactic acid bacterium whose genome was sequenced [1], and it is extensively used for production of different metabolic products and recombinant proteins [reviews in [2-4]]. As this bacterium is generally recognised as safe (GRAS), there has been increasing interest in its use as a live vector for mucosal delivery of therapeutic proteins, including nasal and gastrointestinal vaccines [5,6]. However, there exists a remarkable lack of knowledge about the peculiarities of *L. lactis *metabolic regulation, especially regarding amino acid metabolism. There are several defined media designed for *L. lactis *[7-9], however, these are unbalanced and concentrations of individual amino acids are quite high, making their consumption measurements inaccurate as utilization by the cells is small compared to the total content. Lack of reliable information on consumption patterns and regulation of amino acid metabolism hinders design of cheaper balanced complex media and optimization of bioprocesses.

Systems biology approaches where 'omics' methods are combined with advanced cultivation methods, computational and mathematical models form a solid platform for elucidating quantitative peculiarities of metabolism and its regulation in microorganisms. Transcriptome and proteome expression in *L. lactis *have been measured and compared several times in various phases of batch cultivations [10,11]. A multi-omics study where *L. lactis *was cultivated at steady state conditions was carried out by Dressaire et al. [12,13]. They characterized *L. lactis *at the transcriptome level in isoleucine limited chemostat cultures, calculated translation efficiencies based on proteome and transcriptome levels, and showed that energy costs associated with protein turnover in cells are bigger at low growth rates in comparison with higher ones.

To provide more comprehensive knowledge about amino acid metabolism in *L. lactis *we developed a new medium, which allowed studying quantitative patterns of amino acid consumption. To further link amino acid metabolism with the overall physiological state of cells, growth rate dependent trancriptomes, proteomes and extracellular metabolomes were measured and studied together with carbon, nitrogen and ATP, redox balance analyses. *L. lactis *was cultivated in accelerostat (A-stat) continuous cultures as this method allows acquisition of vast amount of data on quasi steady state growing cells and precise determination of growth characteristics, especially investigation of dependences of growth characteristics on residual concentrations of growth limiting substrate (e.g. glucose) which determines the specific growth rate of cells (μ).

## Results

### *L. lactis *growth characteristics

**Figure 1:**
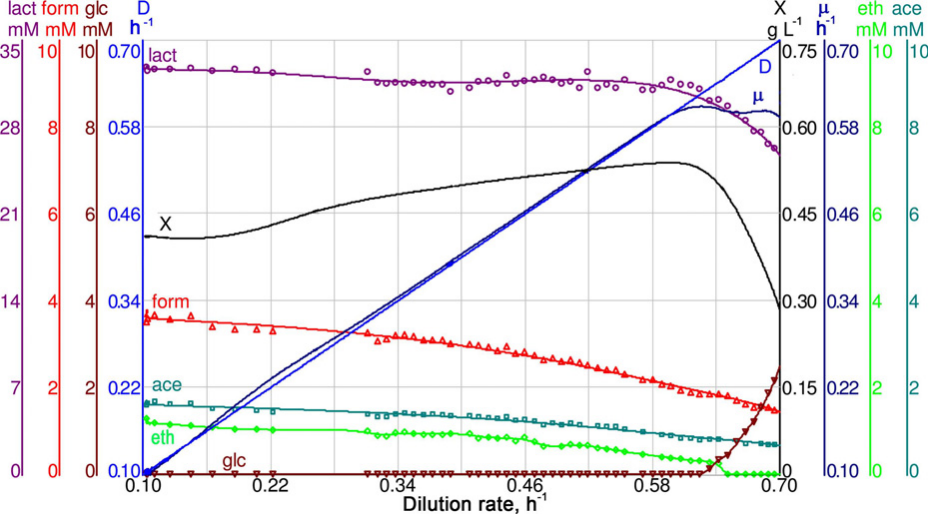
**Typical A-stat cultivation, where dilution rate dependent metabolism of *L. lactis *is illustrated**. D - dilution rate (h^-1^); X - biomass concentration (g (dry cellular weight) L^-1^); μ - specific growth rate (h^-1^); lact, form, glc, eth, ace - lactate, formate, glucose, ethanol, acetate concentration in bioreactor, respectively (mM). D, μ and X are monitored constantly; metabolite concentrations are measured with a frequency of approximately 0.01 h^-1^.

*L. lactis *was cultivated in A-stat culture where after stabilisation in chemostat at dilution rate 0.10 h^-1^, specific growth rate (μ) was smoothly increased until the maximal μ (μ_max_) was reached at 0.59 ± 0.02 h^-1 ^(average value of five independent experiments ± standard deviation; Figure 1). To obtain higher precision in the determination of amino acid consumption patterns, concentrations of most amino acids in the growth medium were reduced *ca *3 times compared to the chemically defined medium (CDM) [14], exceptions being arginine and glutamine, whose concentrations were increased in the medium to avoid amino group shortage during the growth (see Methods). The residual glucose concentration remained below detection limit (<0.1 mM) between μ 0.10 h^-1 ^and 0.59 ± 0.02 h^-1 ^in all five independent experiments. It is important to note that constant protein content (45 ± 2% of cell dry weight) and constant amino acid composition of the protein fraction was observed in the full range of μ from 0.10 to 0.55 h^-1 ^(Additional file 1, Table S1). RNA content increased from 6.5 ± 1.0% to 9.5 ± 1.5% in cell dry weight in between the latter μ values. The biomass yield per consumed carbon (Y_XC_) increased from 0.13 ± 0.00 to 0.17 ± 0.01 C-mol_biomass _C-mol_carbon_^-1 ^when μ was raised from 0.20 ± 0.02 h^-1 ^to 0.52 ± 0.04 h^-1 ^(Additional file 2, Table S1). It was realized by decrease of by-product formation per biomass from 89.6 to 62.3 mmol gdw^-1 ^(sum of Y_lact_, Y_ace _and Y_eth_, Additional file 2, Table S1). Corresponding yield of these by-products (lactate, acetate, ethanol) per consumed glucose decreased from 2.05 to 1.88 mol_products _mol_glc_^-1^, with lactate yield per consumed glucose Y_lg _= 1.83 ± 0.03 mol_lact _mol_glc_^-1 ^remaining constant. As by-product formation exceeded maximal possible yield (2 mol mol^-1^) per consumed glucose at growth rates below 0.4 h^-1 ^(Additional file 1, Table S2) it indicated that part of the amino acids should have been catabolised to pyruvate and eventually to by-products. The overall consumption of amino acids decreased from 12.5 ± 0.5 mmol gdw^-1 ^to 9.3 ± 0.3 mmol gdw^-1 ^with increasing μ (Additional file 2, Figure S1), exceeding two to three times that required for synthesis of proteins in biomass (4.2 ± 0.1 mmol gdw^-1^, Additional file 1, Table S1), and constituting always 21 ± 1% (52 to 39 C-mmol gdw^-1^) of all the total carbon utilised by cells throughout the μ range studied.

For proof of principle, a chemostat experiment was carried out at a dilution rate of 0.45 h^-1 ^and the data obtained were compared with the data obtained at the same μ value in A-stat experiments. The measured substrate and product yields in chemostat culture had values in the range of presented standard deviations for A-stat data (Additional file 2, Table S2) which shows that quasi steady state data from A-stat is comparable to chemostat.

### Amino acid consumption profiles

**Figure 2:**
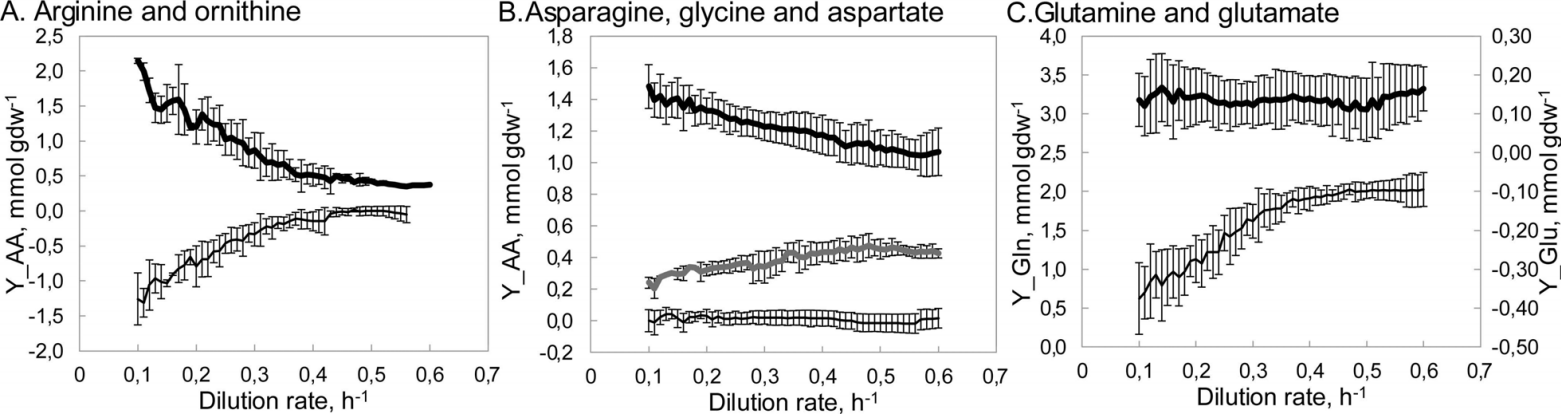
***L. lactis *dilution rate dependent amino acid consumptions (mmol gdw**^**-1**^**) for (A) arginine (thick line) and ornithine (thin line); (B) asparagine (thick line), glycine (dashed line) and aspartate (thin line); (C) glutamine (thick line) and glutamate (thin line)**. Negative numbers on chart represent production. Refer to Additional file 2, Figure S2 for consumption yields of all amino acids.

Based on amino acid concentrations in the cultivation broth, consumption patterns (mmol_AA _gdw^-1^) for all the 20 amino acids were calculated (Figure 2 and Additional file 2, Figure S2). The most abundantly consumed amino acid throughout the μ range studied was glutamine (Additional file 2, Figure S2). Asparagine, arginine, serine, threonine, alanine, leucine, isoleucine and cysteine were the next most intensively consumed amino acids which consumption exceeded notably the amounts necessary for biomass formation. Lysine, phenylalanine and valine were consumed in slightly higher amounts than needed for biomass production. Consumption of aspartate, histidine, and proline were in the range of measurement errors, hence, their consumption can be considered minimal or nonexistent. It has been shown that the latter amino acids are non-essential for the growth of *L. lactis *[8].

In more detail, specific growth rate dependent consumptions of asparagine, threonine and cysteine per biomass were constant in the μ range of 0.10 - 0.20 h^-1^, but decreased 30 to 40% from μ = 0.20 h^-1 ^until μ_max _value (Figure 2 and Additional file 2, Figure S2). Consumption of arginine decreased rapidly in the μ range of 0.10 - 0.35 h^-1 ^from 2.15 ± 0.04 mmol gdw^-1 ^and levelled at 0.44 ± 0.07 mmol gdw^-1 ^at higher growth rates (Figure 2) - at an amount greater than necessary for biomass production (0.20 ± 0.02 mmol gdw^-1^). Decreasing trends in the μ range 0.10 - 0.35 h^-1 ^were observed for the production of ornithine and for the production of the only amino acid produced - glutamate. Glycine was the only amino acid which consumption increased during increasing μ (Figure 2), however, its consumption was always lower than its need for biomass formation. Consumption of other amino acids (Gln, Ile, His, Leu, Lys, Met, Phe, Tyr, Trp, Val) did not change significantly throughout the studied μ range, indicating also a more efficient use of amino acids at higher μ values as growth yields based on carbon and nitrogen consumption increased.

### Carbon, nitrogen and ATP balances

Carbon recovery which was calculated based on glucose and amino acid consumptions, product and biomass formation was 100 ± 2% over the entire μ range (Additional file 2, Figure S3). However, nitrogen recovery, calculated based on amino acid utilization and ornithine, glutamate and biomass formation, was 55 ± 3% (Additional file 2, Figure S3). Amino acids were the main nitrogen source in the medium, comprising more than 99% of the consumed nitrogen by the cultivated bacterium. Based on amino acid utilization, the total consumption of nitrogen decreased from 22 to 14 mmol gdw^-1 ^between the μ range 0.10 - 0.59 ± 0.02 h^-1^. On the basis of monomer composition, N-molar content in the biomass was found to be constant at 7.2 mmol gdw^-1 ^during the studied μ range. Concomitantly, nitrogen incorporation into the biomass increased from 33 to 50% from total consumed nitrogen in amino acids with increasing μ. The rest of nitrogen (50-67%) could have been metabolised through arginine deiminase (ADI) pathway, by excreting other amino acids (glutamate, aspartate) or through deamination reactions (ammonium). Activity of the ADI pathway decreased in the μ range 0.10 - 0.35 h^-1 ^and nitrogen excretion to ornithine and synthesis of exogenous NH_3 _declined from 4.7 to 0.5 mmol gdw^-1 ^(21 to 4% from total nitrogen consumed) in the above μ range. In addition, 0.4 to 0.06 mmol gdw^-1 ^of nitrogen was excreted as glutamate and 0.1 mmol gdw^-1 ^through transamination reactions with the formation of the following compounds detected and quantified by mass-spectrometry: 4-hydroxyphenylpyruvic acid, hydroxyphenyllactic acid, 2-hydroxy-3-methylbutyric acid, 2-hydroxyisocaproic acid and L-3-phenyllactic acid from tyrosine, phenylalanine or branched chain amino acids (data not shown). The left-over of consumed nitrogen was 9.5 - 6.6 mmol gdw^-1 ^(contributing 44 - 48% from total nitrogen) in the μ range of 0.1 - 0.6 h^-1^. This nitrogen must have been excreted as NH_3 _if the excess of consumed amino acids not incorporated into protein fraction of biomass would have been converted to pyruvate. The latter assumption is supported by the fact that the carbon was fully recovered during the growth. Reduction of carbon and nitrogen wasting led to the increase of the biomass yields based on carbon (including glucose and amino acids) and nitrogen consumption 1.3 and 1.5 times, respectively (from 0.12 to 0.15 C-mol C-mol^-1 ^and from 0.33 to 0.50 N-mol N-mol^-1^), in parallel with the increase of μ from 0.10 to 0.59 ± 0.02 h^-1^.

Based on biomass monomer composition and the stoichiometry of ATP, NAD(P)H and central metabolites for monomer production, μ dependent ATP and NAD(P)H balance calculations were carried out (Additional file 1, Tables S3-S5). Calculations indicated that more ATP was produced than necessary for biomass formation. Presumably the ATP synthesized in excess was wasted in futile cycles. Calculated energy spilling was constant at 60 mmol ATP gdw^-1 ^in the range of the μ 0.10 - 0.15 h^-1 ^and decreased afterwards to 36 mmol gdw^-1 ^at μ_max_, indicating that the metabolism was the most efficient near μ_max _conditions (Additional file 1, Table S5). Similarly calculated NAD(P)H misbalance (spilling) decreased from 3.5 mmol gdw^-1 ^at low growth rates to 0 mmol gdw^-1 ^at specific growth rate >0.45 h^-1 ^(Additional file 1, Table S5). However, latter improvement of balance is inside the range of errors of lactate measurements (as lactate dehydrogenase is the main NAD regeneration reaction in lactic acid bacteria). Therefore a conclusion that redox balance was maintained throughout the studied growth conditions should be drawn.

### Transcriptome and proteome response

**Figure 3:**
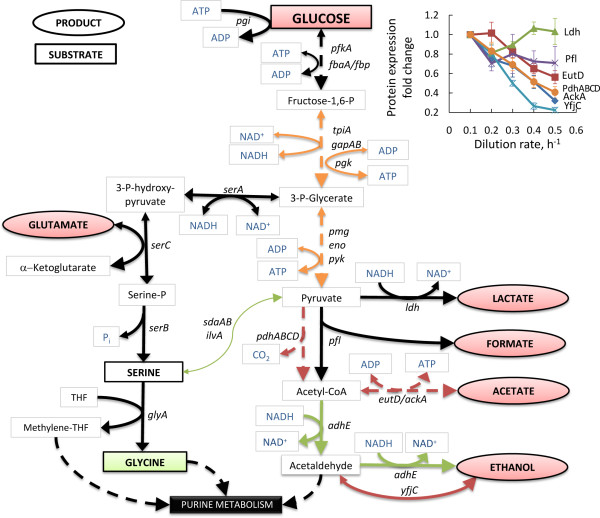
**Overview of central carbon metabolism in *L. lactis *at various specific growth rates (μ)**. Black and capitalised metabolites were measured extracellular. Measured metabolites in boxes/ellipses were consumed/produced, respectively. Red/green/white background represents decrease/increase/no change, respectively, in metabolite consumption or production with increasing μ. Red arrows indicate decrease, green arrows increase and black arrows no significant change in transcriptome and proteome expressions when μ 0.5 h^-1 ^is compared with μ 0.1 h^-1^. Orange arrows represent increase only at transcriptome level with increasing μ. Arrowheads indicate the assumed reaction directions. More specific protein expression fold changes are illustrated on chart.

**Figure 4:**
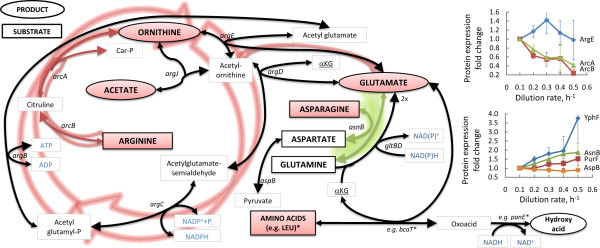
**Overview of arginine and glutamine metabolism in *L. lactis *at various specific growth rates (μ)**. Black and capitalised metabolites were measured extracellular. Measured metabolites in boxes/ellipses were consumed/produced, respectively. Red/white background represents decrease/no change, respectively, in metabolite consumption or production with increasing μ. Red arrows indicate decrease, green arrows increase and black arrows no significant change in transcriptome and proteome expressions when μ 0.5 h^-1 ^is compared with μ 0.1 h^-1^. Arrowheads indicate the assumed reaction directions. Underlined metabolites exist several times on chart. More specific protein expression fold changes are illustrated on chart. Proteins PurF and YphF, represented only on charts, are involved in purine metabolism and converting glutamine to glutamate. THF - tetrahydrofolate; αKG - α-ketoglutarate; Car-P - carbamoyl-phosphate, * - represents example pathway components from literature [38,39].

Transcriptomes and proteomes at four different quasi steady state μ values (0.17, 0.24, 0.44, 0.52 h^-1^) were compared to steady state μ = 0.10 h^-1 ^(additional info in Methods). Changes in gene and protein expression levels for the most relevant reactions between μ 0.52 and 0.10 h^-1 ^are illustrated on Figure 3 and 4; a full list of measured gene and protein expression changes at various μ values can be found in Additional file 3. In this section we discuss changes of mRNA and protein expressions significant with *P *value ≤ 0.05 for μ 0.52 ± 0.03 h^-1 ^*vs*. 0.10 h^-1^.

Mannose uptake genes *ptnAB*, which are responsible for glucose transport in *L. lactis*, and *ptsI *were up-regulated 2.1 to 4.3-fold at the transcriptome level at higher growth rates (above 0.44 h^-1^). However, corresponding enzymes did not show any remarkable change in the same growth rate range as measured in the proteome. Transporter genes for additional sugars (not present in our medium) like galactose (by *galE*) and cellobiose (by *ptcABC *and *yidB*) were 1.8 to 2.9-fold down-regulated at higher specific growth rates at the transcriptome level, whereas a 2.2- to 2.8-fold repression of PtcAB was measured for proteome. This down-regulation is known to be the consequence of carbon catabolite repression which is extensively studied also in other bacteria like *E. coli *and *B. subtilis *[15,16].

Expression in the upper part of glycolysis did not change significantly during increase of μ. However, the lower part of glycolysis (from *fbaA *to *eno*) was 1.8- to 4-times up-regulated at the transcriptome level, but only Pmg showed significant 1.6-fold up-regulation at the proteome level at the growth rates higher than 0.44 h^-1 ^(Figure 3). The pentose phosphate pathway showed a 1.3- to 2.0-fold down-regulation in genes *deoBC, rpiA, zwf, tkt, ywcC *(Additional file 3), which might be explained by a lower NADPH requirements at higher μ conditions. Despite the down-regulation of pentose phosphate pathway, genes encoding proteins involved in purine and pyrimidine metabolism were up-regulated. Moderate, 1.5- to 3.0-fold up-regulation both at the transcriptome and proteome level of the operon PurABEFLMQ was observed. With the increase of purine and pyrimidine metabolism, the need for amino group transfer from glutamine should have been also increased with rising specific growth rate. In agreement with this, expression of the genes in the first steps of purine and pyrimidine synthesis, *purF *increased and *carAB *remained constant respectively, with the increase of μ. High glutamine availability was maintained presumably by increased expression of glutamine transporter (*glnQP*) and glutamine synthetase (*glnA*).

Considering pyruvate metabolism, decreased acetate production was in accordance with the significant down-regulation of genes *eutD *and *ackA2 *and their corresponding enzymes (see Figure 3). However, decreased production of formate and lactate seemed not to be regulated similarly with acetate - Pfl and Ldh showed no major changes neither in gene nor protein expression levels confirming that Ldh is regulated rather by the NADH/NAD^+ ^ratio than by transcription and/or translation, as proposed in literature [17]. Although ethanol production decreased, AdhE expression increased 7.3- and 1.8-fold in transcriptome and proteome analysis, respectively. This might be related to the incorporation of ethanol formation pathway intermediate, acetaldehyde, to acetyl-CoA synthesis from deoxyribose. Pyruvate dehydrogenase subunits (PdhABCD) were 2- to 3-fold down-regulated at both levels (Figure 3).

It is well known, that *L. lactis *can direct part of the consumed (or *de novo *synthesised) serine into pyruvate by *sdaA *and *ilvA *- this flux could form up to 10% of overall pyruvate flux [18]. In the current study, these noted genes were 1.4- to 2.2-fold up-regulated comparing μ = 0.50 to μ = 0.10 h^-1^. In concordance with the sharp decrease of arginine consumption from μ 0.10 h^-1 ^up to μ 0.35 h^-1^, the 2.3- to 4.5-fold decrease in protein expression of ArcAB, which converts arginine to ornithine, was observed during the increase of μ (Figure 4).

## Discussion

### Carbon balance and growth efficiency

Growth conditions have a strong influence on specific growth rate (μ), macromolecular composition of biomass (*i.e*. ribosomal content) and cell size of microorganisms [18,19]. In this study, a gradual change to more efficient carbon metabolism with the increase of μ was observed for *L. lactis *(Figure 1). The first shift in *L. lactis *metabolism took place at μ 0.20 ± 0.02 h^-1^, when biomass yield (Y_XC_) per consumed carbon started to increase. Thirty percent increase with the increase of μ from 0.10 to 0.60 h^-1 ^was achieved by reduction of fermentation by-products synthesis (acetate, formate, ethanol). Concomitantly to the increase of biomass yield, calculated ATP balance showed decreased energy spilling. It has been postulated that higher energy spilling at lower μ conditions could be caused by greater costs of turnover of macromolecules and sensory molecules, establishment of ion gradients across the cell membrane *etc *[20]. Dressaire et al. [12] calculated the degradation rates for proteins and found that protein median half-lives were *ca *10-fold shorter at μ = 0.10 h^-1 ^than at μ_max_. As ATP is consumed during protein degradation [21] this non-growth related expenditure might form a higher proportion of the total energy synthesized at lower μ conditions than at higher growth rates.

### Nitrogen metabolism

With the increase of specific growth rate from 0.10 to 0.60 h^-1 ^biomass yield Y_XN _increased 1.5 times showing that cells used nitrogen more effectively for biomass production. The most important amino acid that plays role in the observed reduction of nitrogen wasting was arginine (arginine consumption decreased from 1.5 to 0.5 mmol gdw^-1 ^with increase of μ from 0.1 to 0.35 h^-1^). Throughout the μ range studied, arginine consumption was 0.3 to 1.3 mmol gdw^-1 ^higher than spent for biomass synthesis and majority of the consumed arginine was transformed to ornithine (0.05 to 1.2 mmol gdw^-1^), especially at lower specific growth rates, which indicates energy limitation of cells. However, not all arginine left over from calculated requirements for biosynthesis (0.1 to 0.25 mmol gdw^-1^) was converted to ornithine. Based on annotated network of *L. lactis *there is no route for consumption of ornithine other than that leading to the synthesis of glutamate (mediated by ArgCDJFG, which were reduced with increase of specific growth rates especially after 0.4 h^-1^). Although the mechanisms of arginine overconsumption in addition to ornithine production are not known, correlation between ornithine production and glutamate synthesis was 0.99, which shows that these syntheses were most probably coupled. Production of glutamate has also been observed before, when both glutamine and glutamate were present in the cultivation medium [8,22].

Nitrogen wasting through glutamine metabolism was not decreased during the increase of specific growth rate. Glutamine, the most consumed amino acid (glutamine consumption covers 30 to 50% of total nitrogen consumed, at μ 0.10 and 0.60 h^-1^, respectively), is used for synthesis of biomass proteins and it is the donor of amino groups in purine, pyrimidine and in aminosugar production pathways (glutamine and glutamate requirements for transamination reactions in aminosugar and nucleotide synthesis was in average 1.35 mmol gdw^-1^). It should be noted that glutamine synthetase (*glnA*) was highly expressed (having array spot intensity values up to four times higher than these of average values of all genes) and increased with increase of μ in parallel to high consumption of the amino acid. Although we cannot argue over the direction of reactions on the basis of our experimental data, it could be assumed that maintenance of high intracellular concentrations of glutamine in the cells in the result of intense synthesis and consumption flows might be necessary to keep the transfer of amino group effective.

The third biggest part of nitrogen wasting could be associated with the consumption of asparagine, which was reduced from 1.4 to 1.1 mmol gdw^-1 ^with increase of μ from 0.10 to 0.60 h^-1^. Asparagine and aspartate (which was not consumed and therefore should have been produced from asparagine) are required for syntheses of nucleotides (in average 0.35 mmol gdw^-1^) and proteins (in average 0.4 mmol gdw^-1^). It was shown that 0.35 to 0.65 mmol gdw^-1 ^of asparagine was not used for biosynthesis. Asparagine can be metabolised through asparaginase (*ansB*) - however its expression was in the range of threshold values in the mRNA array and corresponding protein was not detected. Instead of that the high expression (array spot intensity values up to seven times higher than these of average values of all genes) of asparagine synthetase (*asnB*), which expression even increased with increase of specific growth rate was observed. Similarly to glutamine it could be assumed that overconsumption of asparagine and high expression of the relevant synthesis genes might be necessary to keep the transfer of amino group effective. Energetically transport of asparagine in *L. lactis *is much more efficient than aspartate [23], moreover, asparagine is probably preferentially directed towards the production of aspartate [24,25]. Surplus of aspartate in its turn can be directed into pyruvate by AspB (Figure 4).

The role of other amino acids (other than glutamine, arginine and aspartate) in nitrogen wasting can be considered minimal as over-consumptions (amounts greater than necessary for biomass production) of these amino acids were below 0.2 mmol gdw^-1 ^(cysteine, serine, threonine) or 0.1 mmol gdw^-1 ^(all other not mentioned above).

### Omics comparison

**Figure 5:**
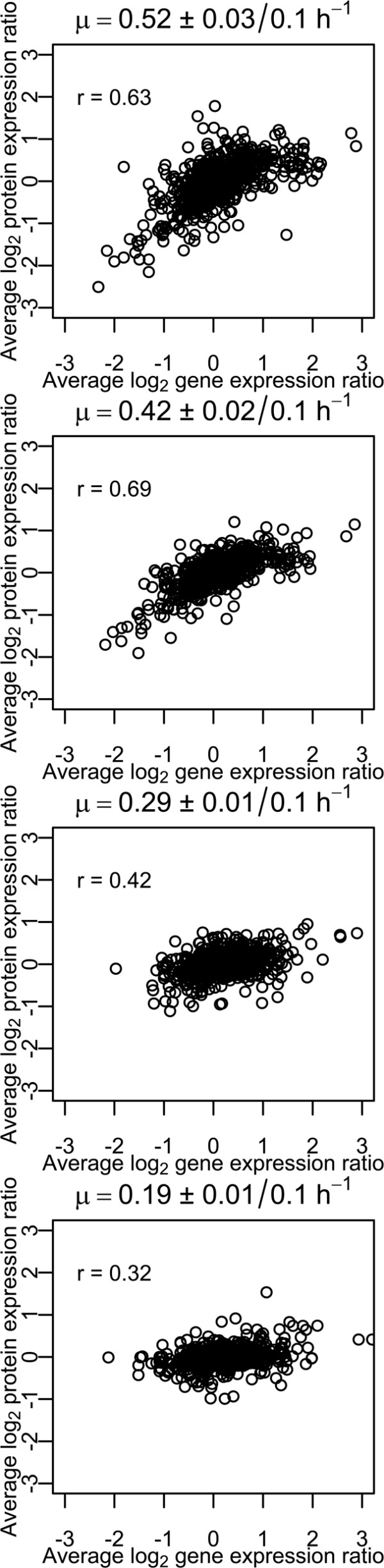
***L. lactis *transcriptome and proteome correlation at various specific growth rates**. "R" value on chart represents Pearson coefficient. Six hundred proteins, with a standard deviation less than 30% and their corresponding genes are indicated on a graph.

Good correlation with a Pearson coefficient up to 0.69 was observed between 600 measured protein and gene expression data (Figure 5). An interesting phenomenon was seen at μ values 0.52 ± 0.03 h^-1 ^and 0.42 ± 0.02 h^-1 ^compared to 0.10 h^-1^: a large amount of genes up-regulated at the transcriptome level showed only small or no change at the proteome level (Figure 5). The vast majority of members in this group were related to ribosomal subunits (74% from all detected ribosomal proteins), as well as lower glycolysis (FbaA, GapB, Pgk, Eno) and amino acid or peptide transport (BusAB, GlnPQ, GltPS, OptCD, PepCPX, PtnABD, PtsHI). Up-regulation at the transcriptome level and no significant change at proteome level during anaerobic growth of *L. lactis *in lower part of glycolysis have also been noticed before [11,12]. Despite the relatively good correlation between the transcriptomic and proteomic data, several important regulations were observed only at trancriptome level. The data obtained indicated importance of taking into account the possibility of allosteric regulation, and carrying out measurements of fluxome in addition to transcriptome and proteome to fully characterize regulation of metabolic pathways.

By scanning the entire range of specific growth rates using A-stat experiments, it is possible to continuously monitor the steady state metabolism using on-line sensors or frequently collected samples for at-line analyses. Reproducibility of growth characteristics in A-stat were compared with chemostat at μ 0.45 h^-1^. All measured substrate consumption and product formation yields (including amino acids) remained within mentioned standard deviation ranges indicating the accordance of quasi steady state and steady state data (Additional file 2, Table S2). Recently, similar comparisons at the global transcriptome level were conducted with *E. coli *achieving very good correlation with a Pearson coefficient up to 0.96 [26]. In both studies, it was shown that the A-stat cultivation technique allows precise monitoring the sequence of metabolic switch points.

## Conclusions

Distinct ratios of glucose and amino acids in the growth media are very important for biomass yield optimization as carbon and nitrogen metabolism are tightly coupled in *L. lactis*. High biomass yields are crucial for producing vaccines using microorganisms and nutrient limitations can strongly affect achieving the desired results. As was shown in this study, some amino acids were consumed in large amounts (glutamine, asparagine, arginine) and more efficient growth might not be achieved by insufficient supply of these compounds. There have been several attempts to optimize the media for lactococci using a single omission technique [7,8], however, a systematic approach taking into account that amino acid requirements depend on environmental conditions (*e.g*. at various μ values) has not yet been fully realized as it is difficult using only batch cultivation. The current work combining systematic continuous cultivation approach with omics methods is therefore of high value for better media design, as well as for understanding principles of metabolism of the bacteria.

Using steady state cultivation methods and a systems biology approach for characterisation of *L. lactis *metabolism, it was possible to demonstrate a shift to more efficient metabolism at higher growth rates by increasing the biomass yield, change towards homolactic fermentation, and decreasing the flux through alternative energy generation pathways with lower efficiency than glycolysis e.g. acetate formation and the ADI pathway.

This study demonstrates the necessity of using strictly controlled continuous cultivation methods in combination with a multi-omics approach and element balance calculations to gain quantitative understanding of the regulation of complex global metabolic networks, important for strain dependent media optimisation or the design of efficient producer cells. However, questions about rationale of 2-3 times over-consumption of amino acids by cells and principles of properly balanced media remain to be answered in full in the future studies.

## Methods

### Microorganism and medium

The strain used throughout these experiments *Lactococcus lactis *subsp. *lactis *IL1403 was kindly provided by Dr. Ogier from INRA (Jouy-en-Josas, France). Inoculum was prepared using a lyophilized stock culture stored at -80°C which was pre-grown twice on the cultivation medium. Chemically defined medium with a reduced amino acid concentrations were developed especially for better detection of amino acids. Media contained 70% GIBCO™ F-12 Nutrient Mixture (Invitrogen Corporation, Carlsbad, CA) and 30% modified CDM (composition in [27]). This combination gave the best trade-off for growth yield and maximal growth rate. Composition of the final medium was as follows (mg L^-1^): limiting substrate D-Glucose - 3500; L-Alanine - 78; L-Arginine - 185; L-Asparagine - 74; L-Aspartic acid - 72; L-Cysteine - 64; L-Glutamic acid - 70; L-Glutamine - 132; Glycine - 58; L-Histidine - 60; L-Isoleucine - 102; L-Leucine - 207; L-Lysine - 158; L-Methionine - 41; L-Phenylalanine - 86; L-Proline - 92; L-Serine - 163; L-Threonine - 76; L-Trypthophan - 16; L-Tyrosine - 29; L-Valine - 107; Biotin - 0.305; Choline chloride - 9.8; D-Pantothenate - 0.65; Folic Acid - 1.21; Niacinamide - 0.325; Pyridoxine hydrochloride - 0.642; Riboflavin - 0.326; Thiamine hydrochloride - 0.51; Vitamin B12 - 0.98; i-Inositol - 12.6; CaCl_2 _- 28; CuSO_4 _× 5H_2_O - 0.272; FeSO_4 _× 7H_2_O - 0.71; MgCl_2 _- 58; KCl - 157; NaCl - 5580; Na_2_PO_4 _- 99; ZnSO_4 _× 7H_2_O - 1; Hypoxanthine-Na - 3; Linoleic Acid - 0.1; Lipoic Acid - 0.1; Phenol Red - 0.8; Putrescine × 2HCl - 0.1; Na-Pyruvate - 77; Thymidine - 0.5.

### A-stat cultivations

A-stat cultivations were carried out in a 1 L Biobundle bioreactor (Applikon, Schiedam, the Netherlands) controlled by an ADI1030 biocontroller (Applikon) and a cultivation control program "BioXpert NT" (Applikon) (detailed description in [28], with an addition of an *in situ *OD sensor (model TruCell2; Finesse, San Jose, CA)). Cultivations were carried out under anaerobic conditions (N_2_-environment) with an agitation speed of 300 rpm at 34°C and pH 6.4. Five parallel A-stat experiments were carried out where after a batch phase, constant dilution rate (D = 0.1 h^-1^) was initiated. Culture was stabilised until constant optical density and titration rate, pumping through at least 5 volumes of medium. After achieving steady state conditions, acceleration of dilution rate (a = 0.01 h^-2^) was started. Additionally, a steady state chemostat experiment was carried out at a dilution rate of 0.45 h^-1 ^and results were compared with data collected from the A-stat experiment at the same dilution rate. Average yield and metabolic switch point values with their standard deviations were calculated based on five independent experiments, additionally taking into account chemostat experiment values at a dilution rate of 0.45 h^-1^.

### Analytical methods and growth characteristics

Biomass was constantly monitored by measuring the optical density at 600 nm; biomass dry weight was determined gravimetrically. Biomass correlation constant K was 0.372 ± 0.005 and was not specific growth rate dependent. Levels of glucose, lactate, formate, acetate and ethanol in the culture medium were measured with liquid chromatography (Alliance 2795 system, Waters Corp., Milford, MA), using a BioRad HPX-87H column (Hercules, CA) with isocratic elution of 5 mM H_2_SO_4 _at a flow rate of 0.6 mL min^-1 ^and at 35°C. A refractive index detector (model 2414; Waters Corp.) was used for detection and quantification of substances. The detection limit for the analytical method was 0.1 mM. Samples from culture medium were centrifuged (14,000 × *g*, 4 min); supernatants were collected and analyzed immediately or stored at -20°C until analysis. Free amino acid concentrations were determined from the same sample (analysing frequency *ca *0.02 h^-1^) with an amino acid analyzer (Acquity UPLC; Waters Corp.) according to the manufacturer's instructions. Empower software (Waters Corp.) was used for the data processing. For measuring amino acid concentrations in protein content, biomass was hydrolysed with 6 M HCl for 20 h at 120°C. From hydrolyte, amino acids were determined as free amino acids described above. Protein content from biomass dry cell weight was calculated based on amino acid analysis and, additionally, measured using the Lowry method [29], where bovine serum albumin was used as a standard. For measurement of DNA content in biomass genomic DNA was extracted and measured using instructions of RTP^® ^Bacteria DNA Mini Kit (Invitec, Germany). Detailed protocol for fatty acid quantification is described in [30]. Growth characteristics μ, Y_XS_, Y_Substrate_, Y_Product _were calculated as described previously [27,28]. For consumption calculations, measured medium concentrations were used.

### Carbon, nitrogen and ATP balance calculations

For carbon balance calculations C-molar concentrations of measured substrates, products and biomass were used (biomass C-molar concentration with a value 0.03625 C-mol gdw^-1 ^was calculated based on monomer composition). For nitrogen balance calculations N-molar amino acid consumptions, production of ornithine and glutamate, ADI pathway activity and biomass composition (0.00725 N-mol gdw^-1^) were taken into account.

For calculations of ATP and NAD(P)H balance measured biomass, amino acid, RNA, DNA and fatty acid contents were used. Other necessary data were adapted from literature [31]. Stoichiometry of ATP, NAD(P)H and central metabolites for monomer production were taken from the Kyoto Encyclopaedia of Genes and Genomes database http://www.kegg.jp/, with an assumption that amino acids were not synthesized. Specific calculations are presented in Additional file 1.

### Gene expression profiling

Agilent's DNA microarrays (Santa Clara, CA) were designed in eArray web portal in 8 × 15K format, containing 7 unique probes per target https://earray.chem.agilent.com/earray/. Target sequences for 2234 genes were downloaded from Kyoto Encyclopaedia of Genes and Genomes ftp://ftp.genome.jp/pub/kegg/genes/organisms/lla/l.lactis.nuc.

For microarray analysis, steady state chemostat culture of *L. lactis *IL1403 was used as reference (D = 0.10 h^-1^). Subsequent quasi steady state points from A-stat experiment at specific growth rates 0.52 ± 0.03; 0.42 ± 0.02; 0.29 ± 0.01 h^1 ^in biological duplicates and 0.17 h^-1 ^were compared to the reference sample. Transcript change was considered significant if the *P *value between parallel experiments was less than 0.05.

Total RNA was extracted and quantified, cDNA synthesised and labelled as described previously [27], with minor modification: 11 μg of total RNA was used for cDNA synthesis. Hybridization, slide washing and scanning was performed using standard Agilent's reagents and hardware http://www.chem.agilent.com. Gene expression data was analyzed as described before [27], except global lowess normalization was used. Spots with intensities lower than 100 units in both channels and outliers among technical replicates (according [32]) were filtered. After filtering, seven technical replicates showed average standard deviation <10%. Gene (and protein) expression measurement results are shown in Additional file 3. DNA microarray data is also available at NCBI Gene Expression Omnibus (Reference series: GSE26536).

### Protein expression profiling

For protein expression analysis, the steady state chemostat culture of *L. lactis *IL1403 was used as reference (μ = 0.10 h^-1^). Quasi steady state points at μ = 0.20 ± 0.01, 0.30 ± 0.02, 0.42 ± 0.01 and 0.50 ± 0.01 h^-1 ^were compared with the reference sample. Three biological replicates were analysed.

Samples intended for proteome analysis were collected, washed with PBS *(*0.137 M NaCl, 2.7 mM KCl, 10.0 mM Na_2_HPO_4, _1.4 mM KH_2_PO_4_), flash frozen in liquid nitrogen and stored at -80°C prior to protein extraction.

Proteins were extracted in ice-cold SDS-buffer (100 mM Tris-HCl (pH 6.5), 1% SDS (w/v)). Cells were disrupted as a result of agitating the suspension with glass-beads at 4°C for 30 minutes. After centrifugation for 30 min at 4°C, the supernatant was collected and the protein concentration was determined by 2D Quant kit (Amersham Biosciences, Buckinghamshire, UK) and protein samples were stored at -80°C until further analysis.

Aliquots of 100 μg cloroform/MeOH chloroform precipitated proteins from each sample were processed for labeling with iTRAQ 4plex reagents (Applied Biosystems, Foster City, CA) according to manufacturer's instructions. Briefly, precipitated proteins were dissolved in 0.5 M triethylammonium bicarbonate (TEAB) and 0.1% SDS, disulfide bonds were reduced in 5 mM Tris-(2-carboxyethyl) phosphine (TCEP) for 1 h at 60°C, followed by blocking cycteine residues in 10 mM methyl methanethiosulfonate (MMTS) for 30 min at room temperature, before digestion with trypsin (1:40, enzyme to protein ratio) overnight at 37°C. For labeling, each iTRAQ reagent was dissolved in 70 μl of ethanol and added to the respective peptide mixture. After 1 h incubation at room temperature the reactions were stopped by adding 100 μl milliQ water and incubating for 30 min. All four samples were mixed together and ethanol was removed by drying in a vacuum concentrator (Model 5301, Eppendorf, Cambridgeshire, UK).

The combined peptide mixtures were separated into 10 fractions with a cation exchange cartridge system (Applied Biosystems, Foster City, CA) by different KH_2_PO_4 _concentrations (10-1000 mM) and cleaned by StageTips [33]. All fractions were analyzed twice by LC-MS/MS using an Agilent 1200 series nanoflow system (Agilent Technologies, Santa Clara, CA) connected to a Thermo Scientific LTQ Orbitrap mass-spectrometer (Thermo Electron, San Jose, CA) equipped with a nanoelectrospray ion source (Proxeon, Odense, Denmark). Purified peptides were dissolved in 0.5% formic acid and loaded on self-packed fused silica emitter (150 mm × 0.075 mm; Proxeon) packed with Repropur-Sil C18-AQ 3 μm particles (Dr. Maisch, Germany) using a flow rate of 0.7 μl min^-1^. Peptides were separated with a 180 min gradient from 3 - 40% B (A: 0.1% formic acid, B: 0.1% formic acid/80% acetonitrile) using a flow-rate of 200 nl min^-1 ^and sprayed directly into LTQ Orbitrap mass-spectrometer operated at 180°C capillary temperature and 2.4 kV spray voltage.

Mass spectrometry method combined HCD and CID spectrums as described in Köcher et al. [34]. Briefly, full mass spectra were acquired in profile mode, with mass range from *m/z *300 to 1800 at resolving power of 60000 (FWHM). Up to four data-dependent MS/MS scans with CID and four scans with HCD tandem mass spectrometry experiment triggered from the same precursor ion were acquired in centroid mode for each FTMS full-scan spectrum. CID was carried out with a target signal value of 10 000 in the linear ion trap, collision energy of 35%, Q value of 0.25 and an activation time of 30 ms. HCD-generated ions were detected in the Orbitrap using the target signal value of 10 000, collision energy of 35% and an activation time of 40 ms. Each fragmented ion was dynamically excluded for 60s.

Raw files were extracted to .mgf files by MM File Conversion Tools http://searcher.rrc.uic.edu/cgi-bin/mm-cgi/downloads.py. Each .mgf file was converted to a QuantMerge file [34]. All files from the same sample were merged together. Data generated was searched against *L. lactis *IL1403 NCBI database (22092009) by MassMatrix search tool [35]. A reversed decoy database was used for false positives detection. In all cases, a peptide mass tolerance of 5 ppm was used and fragment ion masses were searched with a 0.6 Da mass window. Two missed cleavage sites for trypsin were allowed. Beta-methylthiolation of a cysteine was set as a fixed modification and oxidation of methionine as a variable modification. Quantification was set as iTRAQ and quantification statistics as arithmetic mean. Only proteins with confidence intervals of more than 95% were allowed for further data analysis (Additional file 3). Proteomic analysis raw data is available at the PRIDE database [36]http://www.ebi.ac.uk/pride under accession numbers 13105-13162 (username: review17185, password: wyd*b6_6). The data was converted using PRIDE Converter http://code.google.com/p/pride-converter[37]. Protein expression change was considered significant if the *P *value between parallel experiments was less than 0.05.

## Competing interests

The authors declare that they have no competing interests.

## Authors' contributions

PJL, KAd, RV designed experiments and conceived the project. PJL, KAl carried out experiments. PJL, RN, LA, KAl contributed in analytics and data analysis. KAd was responsible for mathematical calculations. PJL drafted the manuscript. KAd helped drafting the manuscript. RV, RN, LA edited the manuscript. All authors read and approved the final manuscript.

## Supplementary Material

Additional file 1 **Specific growth rate dependent ATP and NAD(P)H balance calculations for A-stat experiments with *Lactococcus lactis *subsp. *lactis *IL1403**. Click here for file

Additional file 2 **Supplementary figures and tables**. Click here for file

Additional file 3 **Specific growth rate dependent mRNA and protein expression changes from A-stat experiments with *Lactococcus lactis *subsp. *lactis *IL1403**. The expression fold change is given accordingly: sample at respective specific growth rate (quasi steady state) is divided by steady state chemostat sample (0.10 h-1). Average log2 gene and protein expression changes were calculated from "n" number of parallel A-stat experiments. In gene expression analysis spots with intensities lower than 100 units in both channels and outliers among technical replicates (according Rorabacher, 1991) were filtered. In protein expression analysis, proteins identified with a confidence interval more the 95% and appearances in all mentioned parallels are presented. Click here for file
