# Supplementary material for: Multi-omics approach to study the growth efficiency and amino acid metabolism in Lactococcus lactis at various specific growth rates
Source: Microb Cell Fact. 2011 Feb 24;10:12. doi: 10.1186/1475-2859-10-12 (PMC3049130; doi:10.1186/1475-2859-10-12)
Supplement: Additional file 2 — Supplementary figures and tables. [file 1475-2859-10-12-S2.PDF]

Additional file 2

Supporting Information for:

Multi-omics approach to study the growth efficiency and amino acid metabolism in *Lactococcus lactis* at various specific growth rates

Petri-Jaan Lahtvee[a,b], Kaarel Adamberg[b,c], Liisa Arike[b,c], Ranno Nahku[a,b], Kadri Aller[a,b], Raivo Vilu[a,b,]*

[a]Tallinn University of Technology, Department of Chemistry, Akadeemia tee 15, 12618 Tallinn, Estonia

[b]Competence Center of Food and Fermentation Technologies, Akadeemia tee 15b, 12618 Tallinn, Estonia

[c]Tallinn University of Technology, Department of Food Processing, Ehitajate tee 5, 19086 Tallinn, Estonia

E-mail addresses:

PJL: petri@tftak.eu
KAd: kaarel@tftak.eu
LA: liisa@tftak.eu
RN: ranno@tftak.eu
KAl: aller@tftak.eu
RV: raivo@kbfi.ee

*To whom correspondence should be addressed. Tallinn University of Technology, Department of Chemistry, Akadeemia tee 15, 12618, Tallinn, Estonia. Tel: +372 6204831. Fax : +372 6202828. E-mail: raivo@kbfi.ee.

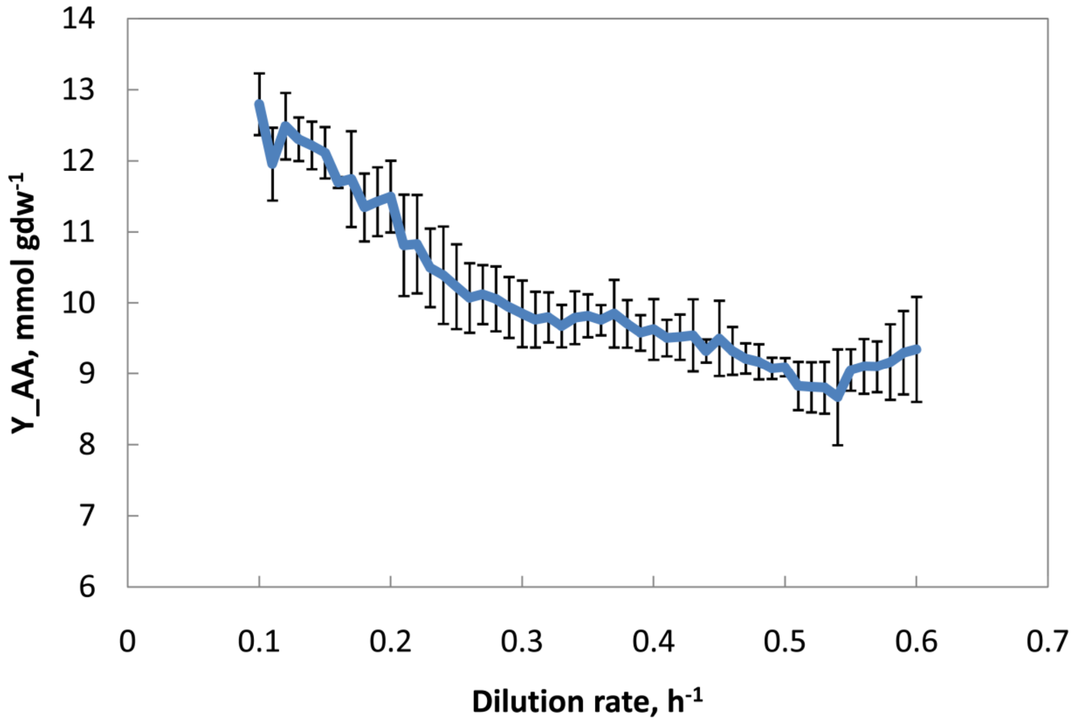

Fig. S1. *Lactococcus lactis* specific growth rate dependent total amino acid consumption profiles. Blue line represents average consumption (mmol gdw^{-1}) from five independent A-stat experiments and error bars represent their standard deviation.

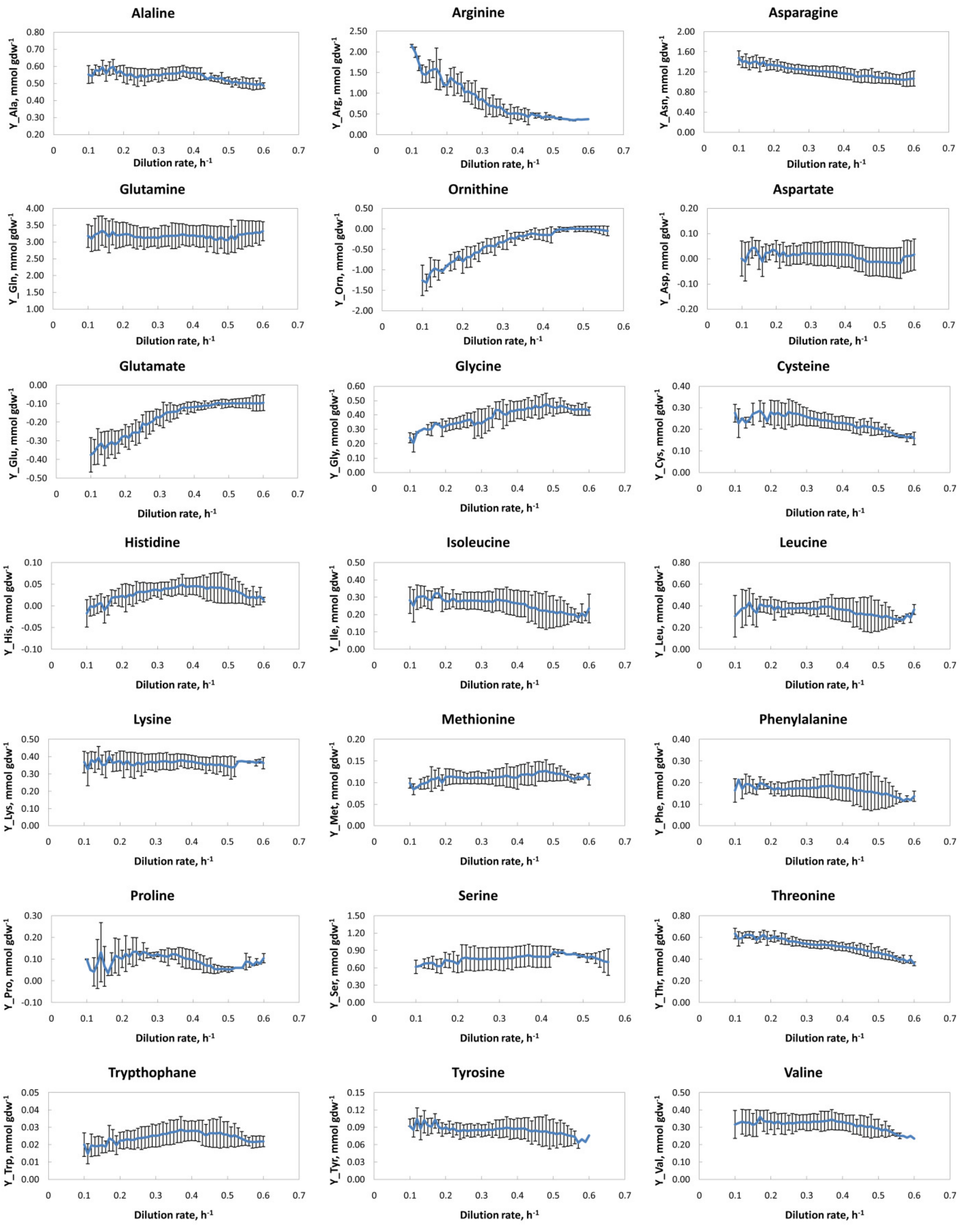

Fig. S2. *Lactococcus lactis* specific growth rate dependent amino acid consumption profiles. Blue line represents average consumption yields (mmol gdw^{-1}) from five independent A-stat experiments and error bars represents their standard deviation. Negative values represent production.

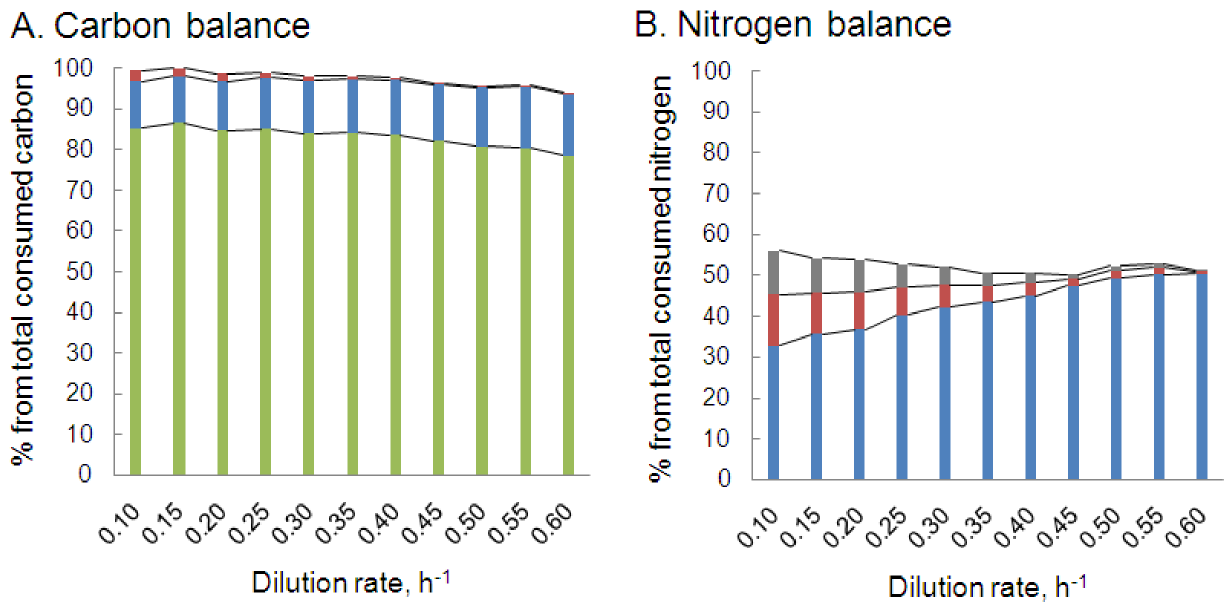

Fig. S3. *Lactococcus lactis* specific growth rate dependent (A) carbon and (B) nitrogen balance. On graph A green represents fermentation products from pyruvate (lactate, formate, acetate, ethanol), blue represents biomass formation and red represents production of ornithine and glutamate. On graph B blue represents biomass formation, red represents production of ornithine and glutamate and grey represents ammonia produced during arginine deiminase pathway.

Table S1. ***Lactococcus lactis*** **specific growth rate dependent biomass yield (Y_{XS}, gdw g_{glc}^{-1}) and lactate (Y_lact), acetate (Y_ace), formate (Y_form) and ethanol (Y_eth) production yields (mmol gdw^{-1}).** Values represent average of five independent A-stat experiments with their standard deviations.

	Average values					RSD				
D	Yxs	Y_lact	Y_ace	Y_form	Y_eth	Yxs	Y_lact	Y_ace	Y_form	Y_eth
h^{-1}	gdw g_{glc}^{-1}	mmol gdw^{-1}				gdw g_{glc}^{-1}	mmol gdw^{-1}			
0.10	0.127	80.63	5.63	8.50	1.71	0.003	4.47	1.53	0.38	1.12
0.11	0.126	81.3	5.58	8.49	1.66	0.002	3.50	1.54	0.28	1.00
0.12	0.127	82.04	4.81	8.60	1.71	0.003	4.28	1.45	0.16	1.18
0.13	0.127	82.03	4.74	8.58	1.65	0.003	3.88	1.34	0.11	1.10
0.14	0.126	82.54	5.34	8.48	1.56	0.005	2.89	1.47	0.11	0.77
0.15	0.126	82.56	5.27	8.45	1.56	0.006	3.35	1.49	0.08	0.75
0.16	0.131	78.42	4.89	8.01	1.49	0.004	4.53	1.08	0.66	0.76
0.17	0.128	79.49	5.07	8.08	1.50	0.002	2.93	1.28	0.42	0.73
0.18	0.132	77.88	4.90	7.90	1.48	0.002	2.91	1.19	0.54	0.72
0.19	0.131	78.51	4.94	7.93	1.51	0.004	3.39	1.30	0.40	0.72
0.20	0.134	76.99	4.40	8.07	1.77	0.005	4.10	1.07	0.55	0.86
0.21	0.132	78.43	4.51	8.05	1.78	0.005	4.10	1.29	0.52	0.81
0.22	0.134	76.95	4.41	7.92	1.76	0.005	3.88	1.20	0.62	0.81
0.23	0.136	76.15	4.38	7.86	1.74	0.005	4.22	1.23	0.61	0.80
0.24	0.138	74.74	4.31	7.74	1.72	0.005	4.06	1.21	0.61	0.79
0.25	0.139	74.16	4.29	7.70	1.7	0.006	4.36	1.25	0.62	0.78
0.26	0.140	72.97	4.23	7.61	1.67	0.006	4.11	1.26	0.61	0.76
0.27	0.140	73.08	4.23	7.63	1.66	0.007	4.75	1.35	0.62	0.73
0.28	0.142	72.04	4.16	7.52	1.62	0.007	4.59	1.33	0.62	0.70
0.29	0.143	71.26	4.08	7.43	1.58	0.008	4.61	1.27	0.66	0.69
0.30	0.144	70.62	4.01	7.33	1.53	0.008	4.7	1.21	0.67	0.66
0.31	0.144	70.91	3.99	7.30	1.49	0.008	5.28	1.25	0.66	0.62
0.32	0.145	70.59	3.89	7.18	1.45	0.008	5.88	1.15	0.63	0.60
0.33	0.146	70.25	3.82	7.06	1.47	0.007	5.94	1.11	0.56	0.63
0.34	0.146	70.54	3.78	7.00	1.50	0.007	5.99	1.13	0.51	0.67
0.35	0.146	70.43	3.73	6.94	1.48	0.008	6.02	1.09	0.50	0.68
0.36	0.147	69.72	3.64	6.8	1.42	0.008	6.16	1.00	0.48	0.66
0.37	0.147	70.06	3.62	6.75	1.39	0.008	6.28	1.07	0.49	0.64
0.38	0.148	69.73	3.56	6.63	1.34	0.009	6.41	1.09	0.51	0.61
0.39	0.150	68.63	3.45	6.44	1.27	0.008	6.05	1.03	0.46	0.59
0.40	0.149	68.96	3.45	6.38	1.22	0.010	6.31	1.11	0.54	0.56
0.41	0.151	67.78	3.33	6.19	1.12	0.009	5.72	1.01	0.45	0.51
0.42	0.152	67.42	3.28	6.07	1.09	0.008	4.98	1.03	0.42	0.51
0.43	0.152	67.07	3.22	5.93	1.07	0.009	4.45	1.06	0.41	0.54
0.44	0.155	65.43	3.06	5.69	1.02	0.008	3.68	0.88	0.28	0.55
0.45	0.153	66.31	3.03	5.64	0.98	0.009	3.97	1.00	0.38	0.53
0.46	0.155	65.44	2.93	5.47	0.91	0.009	3.28	1.14	0.35	0.49
0.47	0.156	64.93	2.87	5.31	0.82	0.009	3.00	1.14	0.41	0.43
0.48	0.158	64.08	2.75	5.09	0.78	0.008	2.57	1.08	0.35	0.43
0.49	0.160	63.19	2.62	4.87	0.77	0.007	2.39	1.00	0.35	0.48
0.50	0.159	63.26	2.60	4.78	0.75	0.007	2.94	1.05	0.44	0.51
0.51	0.165	62.33	2.48	4.6	0.69	0.009	3.13	0.98	0.38	0.49
0.52	0.163	62.52	2.48	4.51	0.64	0.008	3.67	1.04	0.49	0.46
0.53	0.164	62.22	2.40	4.36	0.60	0.009	4.22	1.02	0.45	0.43
0.54	0.165	61.73	2.31	4.21	0.56	0.009	4.86	0.95	0.43	0.42
0.55	0.166	61.07	2.22	4.08	0.50	0.008	5.45	0.88	0.37	0.38
0.56	0.166	60.58	2.17	4.01	0.62	0.008	5.57	0.86	0.3	0.23
0.57	0.169	59.94	2.12	3.93	0.57	0.010	5.39	0.86	0.22	0.24
0.58	0.171	60.14	2.15	3.93	0.53	0.006	4.89	1.00	0.24	0.23
0.59	0.164	61.08	2.25	3.99	0.47	0.010	5.72	1.25	0.48	0.24
0.60	0.168	59.19	2.13	3.84	0.39	0.008	4.63	1.09	0.29	0.34

Table S2. Comparison of chemostat and A-stat data at dilution rate 0.45 h^{-1} in *Lactococcus lactis* cultivations. Individual chemostat experiment values are compared with an average of 5 A-stat exeriment values (at D 0.45 h^{-1}) and corresponding standard deviation values are presented.

	Chemostat	A-stat (± RSD)			Unit
Yxs*	0.15	0.15	±	0.01	gdw g_{glc}^{-1}
Ylg**	1.78	1.82	±	0.03	mol_{lact} mol_{glc}^{-1}
Carbon recovery	98.0	100.7	±	2.2	%
Consumption yields					
Y_Glucose	36.36	36.31	±	1.95	mmol gdw^{-1}
Y_Arginine	0.49	0.46	±	0.06	mmol gdw^{-1}
Y_Alanine	0.53	0.54	±	0.02	mmol gdw^{-1}
Y_Asparagine	1.03	1.11	±	0.13	mmol gdw^{-1}
Y_Aspartate	0.04	0.00	±	0.05	mmol gdw^{-1}
Y_Cysteine	0.18	0.21	±	0.04	mmol gdw^{-1}
Y_Glutamine	3.29	3.17	±	0.31	mmol gdw^{-1}
Y_Glycine	0.46	0.47	±	0.07	mmol gdw^{-1}
Y_Histidine	0.04	0.04	±	0.03	mmol gdw^{-1}
Y_Isoleucine	0.28	0.24	±	0.09	mmol gdw^{-1}
Y_Leucine	0.34	0.33	±	0.14	mmol gdw^{-1}
Y_Lysine	0.35	0.36	±	0.04	mmol gdw^{-1}
Y_Methionine	0.09	0.12	±	0.03	mmol gdw^{-1}
Y_Phenylalanine	0.17	0.17	±	0.08	mmol gdw^{-1}
Y_Proline	0.11	0.07	±	0.03	mmol gdw^{-1}
Y_Serine	0.91	0.88	±	0.03	mmol gdw^{-1}
Y_Threonine	0.51	0.49	±	0.05	mmol gdw^{-1}
Y_Tryptophane	0.02	0.03	±	0.01	mmol gdw^{-1}
Y_Tyrosine	0.08	0.08	±	0.02	mmol gdw^{-1}
Y_Valine	0.31	0.31	±	0.06	mmol gdw^{-1}
Y_tot AA***	9.24	9.50	±	0.53	mmol gdw^{-1}
Production yields					
Y_Lactate	64.83	66.31	±	3.97	mmol gdw^{-1}
Y_Formate	5.37	5.59	±	0.35	mmol gdw^{-1}
Y_Acetate	3.05	3.42	±	1.21	mmol gdw^{-1}
Y_Ethanole	1.11	1.01	±	0.46	mmol gdw^{-1}
Y_Glutamate	0.12	0.11	±	0.02	mmol gdw^{-1}
Y_Ornithine	0.04	0.01	±	0.05	mmol gdw^{-1}

* biomass production yield per consumed glucose

** lactate production yield per consumed glucose

*** total amino acid consumption yield per biomass
